# Soluble Siglec-9 suppresses arthritis in a collagen-induced arthritis mouse model and inhibits M1 activation of RAW264.7 macrophages

**DOI:** 10.1186/s13075-016-1035-9

**Published:** 2016-06-07

**Authors:** Takuya Matsumoto, Nobunori Takahashi, Toshihisa Kojima, Yutaka Yoshioka, Jun Ishikawa, Koichi Furukawa, Kenji Ono, Makoto Sawada, Naoki Ishiguro, Akihito Yamamoto

**Affiliations:** Department of Orthopedic Surgery and Rheumatology, Nagoya University Graduate School of Medicine, 65 Tsurumai-cho, Showa-ku, Nagoya, Aichi 466-8550 Japan; Department of Oral and Maxillofacial Surgery/Protective Care for Masticatory Disorders, Nagoya University Graduate School of Medicine, 65 Tsurumai-cho, Showa-ku, Nagoya, Aichi 466-8550 Japan; Department of Biochemistry II, Nagoya University Graduate School of Medicine, Nagoya, 466-0065 Japan; Research Institute of Environmental Medicine, Nagoya University, Nagoya, 464-8601 Japan

**Keywords:** Rheumatoid arthritis, Collagen-induced arthritis, Siglec-9

## Abstract

**Background:**

The aim of this study was to assess the effects of soluble sialic acid-binding immunoglobulin-type lectin (sSiglec)-9 on joint inflammation and destruction in a murine collagen-induced arthritis (CIA) model and in monolayer cultures of murine macrophages (RAW264.7 cells and peritoneal macrophages) and fibroblast-like synoviocytes (FLS) derived from patients with rheumatoid arthritis.

**Methods:**

DBA/1J mice were immunized with type II collagen. Effects of sSiglec-9 were evaluated using a physiologic arthritis score, histological analysis, serum tumor necrosis factor (TNF)-α concentration, and the proportion of forkhead box P3 (Foxp3)-positive regulatory T (Treg) cells. In vivo biofluorescence imaging was used to assess the distribution of sSiglec-9. Levels of M1 (TNF-α, interleukin [IL]-6, and inducible nitric oxide synthase) and M2 (CD206, Arginase-1, and IL-10) macrophage markers and phosphorylation of intracellular signaling molecules were examined in macrophages, and levels of matrix metalloproteinase (MMP)-1, MMP-3, and MMP-13 were examined in FLS.

**Results:**

sSiglec-9 significantly suppressed the clinical and histological incidence and severity of arthritis. The proportion of Foxp3-positive Treg cells significantly improved and serum TNF-α concentration decreased in vivo. Although sSiglec-9 reduced the expression of M1 markers in macrophages, it did not affect the expression of M2 markers and MMPs in FLS. Nuclear factor (NF)-kB p65 phosphorylation was attenuated by sSiglec-9, and chemical blockade of the NF-kB pathway reduced M1 marker expression in RAW264.7 cells.

**Conclusions:**

In this study, we have demonstrated the therapeutic effects of sSiglec-9 in a murine CIA model. The mechanism underlying these effects involves the suppression of M1 proinflammatory macrophages by inhibiting the NF-kB pathway. sSiglec-9 may provide a novel therapeutic option for patients with rheumatoid arthritis refractory to currently available drugs.

## Background

Rheumatoid arthritis (RA) is a common autoimmune disease characterized by chronic synovial joint inflammation, including synovial hyperplasia, infiltration of inflammatory cells, fibrin deposition, and joint destruction [1]. Proinflammatory cytokines and proteases secreted from synovial tissue alter chondrocyte metabolism and cartilage matrix degradation, leading to cartilage destruction. Recent evidence suggests that the standard treatment strategies, including methotrexate-based conventional disease-modifying antirheumatic drugs (DMARDs) and biological DMARDs, significantly suppress disease activity and joint destruction in patients with RA. However, these strategies target only certain patient populations [2], and thus there is an unmet need for new antirheumatic drugs with novel mechanisms of action.

Synovial cells in the RA synovial membrane consist of synovial fibroblasts, macrophages, and lymphocytes. Macrophages in inflamed synovial tissue play a central role in the sustenance of synovial inflammation and promotion of tissue destruction [3–5]. Macrophages differentiate from peripheral blood monocytes and express adhesion molecules, chemokine receptors, and other surface antigens, as well as cytokines and chemokines that maintain joint inflammation [6–9]. This suggests that macrophages and their products are promising therapeutic targets in patients with RA.

Sialic acid-binding immunoglobulin-type lectins (Siglecs) are type I transmembrane proteins that bind to sialic acid. They are I-type (immunoglobulin [Ig]-type) lectins that belong to the Ig superfamily and are expressed on the cell surface of various immunocytes [10, 11]. Siglecs have a characteristic N-terminal, Ig-like, V-type domain that mediates binding to sialic acid, as well as varying numbers of Ig-like, C2-type domains (2–17), a single transmembrane region, and a cytoplasmic tail (except for Siglec-1 and Siglec-14). Siglec-9 is a member of the CD33-related Siglecs in humans and corresponds to Siglec-E expressed on murine immunocytes. Siglec-9 downregulates both innate and acquired immune responses, and has immunoreceptor tyrosine-based inhibitory motifs in the cytosolic region [12]. As a receptor, Siglec-9 induces the production of low levels of tumor necrosis factor (TNF)-α upon stimulation with lipopolysaccharide (LPS) and enhances the production of the anti-inflammatory cytokine interleukin (IL)-10 in macrophages [13]. Siglec-9 was recently identified as a candidate ligand on leukocytes [14]. 

Matsubara et al. [15] reported that the extracellular domain of Siglec-9 (soluble Siglec-9 [sSiglec-9]) and monocyte chemoattractant protein 1 (chemokine [C-C motif] ligand 2 [CCL2]) induce functional recovery in a rat spinal cord injury (SCI) model. They found that sSiglec-9 exerts anti-inflammatory effects at the spinal injury site by altering macrophage polarization from being M1-dominant (proinflammatory) to M2-dominant (anti-inflammatory). They concluded that sSiglec-9 and CCL2 are inducers of M2 macrophages and promote functional recovery via their anti-inflammatory effects at the SCI site.

In this study, we used a mouse collagen-induced arthritis (CIA) model to investigate the therapeutic effects of sSiglec-9 in arthritis and subsequent joint destruction in vivo. We also conducted in vitro studies to evaluate the anti-inflammatory effects of sSiglec-9 in murine macrophages (RAW264.7 cells and peritoneal macrophages [pMACs]) and human fibroblast-like-synoviocytes (FLS).

## Methods

### Reagents

Recombinant human Siglec-9 and interferon (IFN)-γ were purchased from R&D Systems (Minneapolis, MN, USA). Recombinant human TNF-α was obtained from PeproTech (Rocky Hill, NJ, USA). Complete Freund’s adjuvant (CFA) was purchased from Chondrex (Redmond, WA, USA). Bovine type II collagen was obtained from Seikagaku (Tokyo, Japan).

### CIA model

All animal experiments were performed in accordance with the National Institutes of Health Guide for the Care and Use of Laboratory Animals and with the approval of our institutional animal ethics committee (Division of Experimental Animals, Nagoya University, and Center for Animal Research and Education, Nagoya University).

**Fig. 1:**
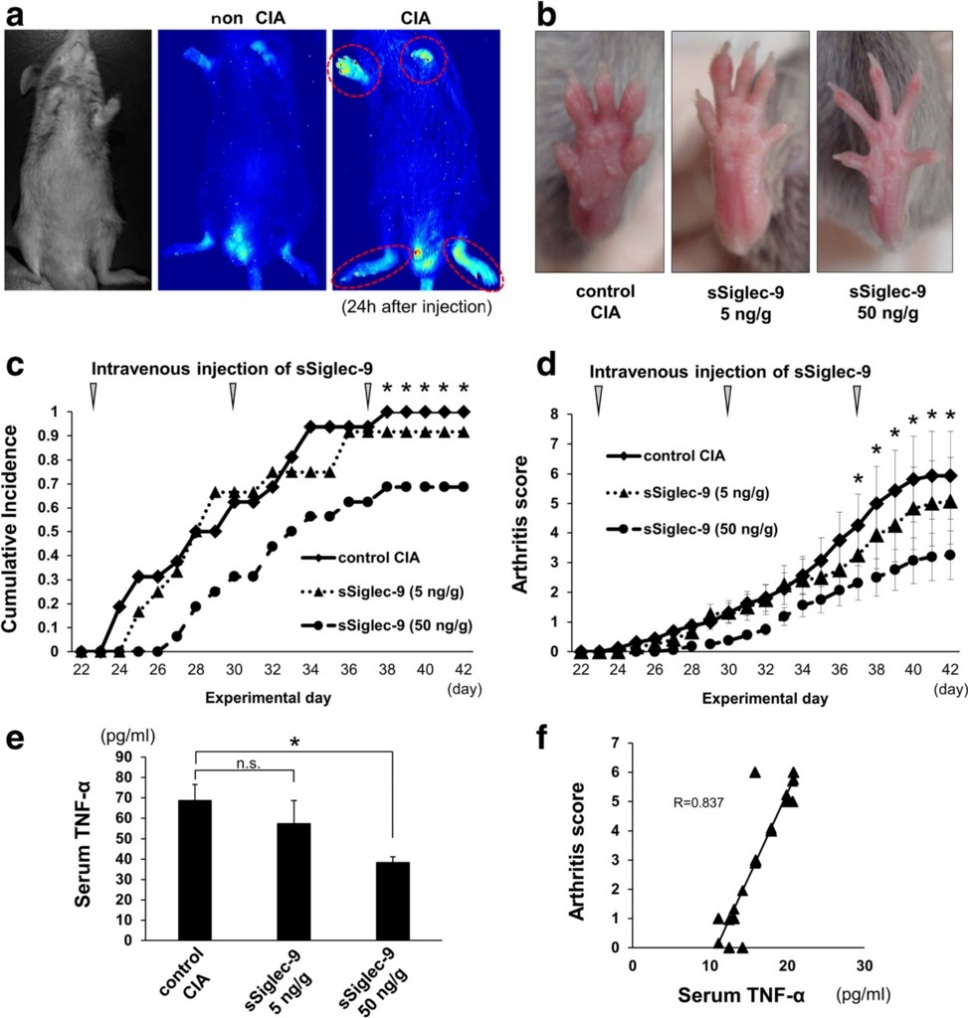
Effects of soluble sialic acid-binding immunoglobulin-type lectin (sSiglec)-9 on arthritis symptoms in mice with collagen-induced arthritis (CIA). **a** Postinjection (24 h, non-CIA versus CIA) images with VivoTag 680 XL-labeled sSiglec-9. Accumulation of fluorescence in CIA joints is denoted by *dashed red circles*. **b** Representative images of hind paws from each treatment group. **c** Cumulative incidence of arthritis in control CIA mice (untreated) and mice treated with different dosages of sSiglec-9. *p < 0.05 for sSiglec-9 50 ng/g versus control (χ^2^ test). **d** Severity of arthritis symptoms as evaluated using the arthritis score in control CIA mice (untreated) and mice treated with different dosages of soluble sSiglec-9. *p < 0.05 for sSiglec-9 50 ng/g versus control (Bonferroni post hoc test). **e** Serum tumor necrosis factor (TNF)-α concentration was significantly decreased in mice treated with 50 mg/g sSiglec-9. *p < 0.05 (Bonferroni post hoc test). **f** Arthritis score and serum TNF-α concentration were strongly and positively correlated (*p* < 0.01, *R* = 0.837 [Pearson product-moment correlation coefficient])

Seven-week-old male DBA/1J mice were purchased from Japan SLC (Hamamatsu, Japan). Mice were immunized subcutaneously at the base of the tail with 100 μl of bovine type II collagen (2 mg/ml) dissolved in 0.01 M acetic acid emulsified 1:1 with CFA; ultimately, each mouse received 100 μg of bovine type II collagen. Mice further received a subcutaneously administered booster tail injection of 50 μg of bovine type II collagen emulsified in incomplete Freund’s adjuvant 21 days later (Fig. 1a).

### sSiglec-9 treatments

Mice (*n* = 23) were randomly allocated to three groups before immunization: a control CIA group (*n* = 8) and two treatment groups with human sSiglec-9 at a dose of 5 ng/g body weight (*n* = 7) or 50 ng/g body weight (*n* = 8). Mice were intravenously administered 100 μl of PBS in the control CIA group or sSiglec-9 (5 or 50 ng/g body weight in a total volume of 100 μl) in the treatment groups weekly from days 23 to 37 (primary immunization day was set as day 0) (Fig. 1a). This schedule was based on preliminary results showing that arthritis almost always developed by day 23 when the above protocol was used (data not shown). These in vivo experiments were performed twice independently with reproducible results.

### Clinical and histological evaluation

CIA was considered to have successfully developed and was regarded as incident when swelling was observed in at least one digit or paw. The severity of arthritis was graded in each paw on a 0- to 3-point scale as follows: grade 0 = normal, grade 1 = swelling of one digit, grade 2 = swelling of two digits, and grade 3 = swelling of the entire paw (Fig. 1e). The cumulative score for four paws of each mouse was used as the arthritis score (maximum score of 12 per mouse) to represent overall disease severity and progression [16]. We calculated the cumulative incidence of arthritis, excluding dropouts, during the experimental period.

On day 42, mice were killed under general anesthesia by systemic perfusion of 4 % paraformaldehyde. The bilateral knee and ankle joints of all mice were dissected and subjected to histological analysis. Histopathological changes in the joints were scored using parameters described in previous reports [17, 18]. Four parameters of histological scores were assessed separately (*n* = 8 each group): inflammation (synovitis), pannus formation, cartilage degeneration, and bone degeneration. We evaluated each parameter as follows: normal = 0, mild = 1, moderate = 2, and severe = 3. The sum of the bilateral scores was calculated (maximum score per mouse = 6). We collected blood from the hearts of mice, and the serum concentration of TNF-α was determined using an enzyme-linked immunosorbent assay (ELISA) assay kit (BioLegend, San Diego, CA, USA) according to the manufacturer’s instructions.

### Immunohistochemical analysis

Ankle joint sections were subjected to immunohistochemical analysis using specific antibodies. The primary antibodies used were mouse anti-TNF-α antibody (1:100 dilution; Abcam, Cambridge, UK), mouse anti-IL-6 antibody (1:100 dilution; Abcam), and antimouse inducible nitric oxide synthase (iNOS) polyclonal antibody (1:1000 dilution; Wako Pure Chemical Industries, Osaka, Japan). Histofine® Simple Stain MAX PO (Nichirei Biosciences, Tokyo, Japan) was used as the secondary antibody. Stained sections incubated without primary antibodies were used as negative controls.

### CD4^+^ T-cell isolation, intracellular staining, and flow cytometry

Spleens from mice were collected for cell preparation and washed twice with PBS. Spleens were minced, and red blood cells were lysed with 0.83 % ammonium chloride. The cells were filtered through a cell strainer and centrifuged at 1300 revolutions per minute for 5 minutes at 4 °C. To purify splenic CD4^+^ T cells, splenocytes were incubated with CD4-coated magnetic beads and isolated using the EasySep isolation kit (STEMCELL Technologies, Vancouver, BC, Canada), and viable CD3^+^CD4^+^ T cells were collected as previously described [19]. Allophycocyanin-conjugated anti-CD25 antibody (eBioscience, San Diego, CA, USA) and anti-forkhead box P3 (Foxp3) antibody (Cell Signaling Technology, Danvers, MA, USA) were used for intracellular staining and flow cytometry. Fluorescence-activated cell sorting analysis was performed using a BD FACSCanto II flow cytometer (BD Biosciences, San Jose, CA, USA). All data were analyzed using FACSDiva software (BD Biosciences).

### In vivo biofluorescence imaging

In vivo biofluorescence imaging was performed to visually analyze the distribution of sSiglec-9. We used CIA mice (on day 40 after first immunization, *n* = 3) and non-CIA (*n* = 3) DBA/1J mice for this imaging study. All mice were fasted overnight before imaging. sSiglec-9 labeled with VivoTag 680 XL (VT) fluorochrome (PerkinElmer, Waltham, MA, USA) was injected intravenously via the tail vein (50 ng/g body weight). VT is an amine-reactive near-infrared fluorochrome used to label biomolecules for in vivo imaging applications (peak excitation 670 nm, peak emission 688 nm. One day before imaging, sSiglec-9 was labeled with VT using the VivoTag protein labeling kit (PerkinElmer) according to the manufacturer’s instructions. All mice were imaged at 6 and 24 h after injection.

Before imaging time points, mice were anesthetized with isoflurane and oxygen, and images were captured with them in the supine position from the dorsal aspect using the Maestro GNIR fluorescence imaging system (PerkinElmer). The filter (excitation and emission) and exposure time settings were determined according to methods described in previous studies [20, 21].

### Isolation of peritoneal macrophages

pMACs were isolated from DBA/1J mice, and 5 % thioglycollate (2 ml; Sigma-Aldrich, St. Louis, MO, USA) was injected into the abdominal cavity to activate pMACs. After 3 days, mice were killed and 10 ml of cold, sterile PBS was injected intraperitoneally. Peritoneal lavage fluid was collected using sterile syringes and centrifuged to obtain the pMAC cell pellet.

### Cell culture

The murine macrophage cell line RAW264.7 was purchased from DS Pharma Biomedical (Osaka, Japan). RAW264.7 cells and pMACs were cultured as monolayers in six-well plates (1 × 10^5^ cells/well), stimulated with 10 ng/ml IFN-γ, and concurrently treated with sSiglec-9 (0–10 ng/ml) for 12 h.

### Isolation and culture of FLS

FLS were isolated from the synovial tissue of five patients with RA who had undergone knee joint replacement surgery. Informed consent was obtained from all patients, and our institutional ethics committee approved the study. Tissue specimens from patients were subjected to monolayer culture according to a previously described protocol [22]. FLS were cultured in six-well plates (1 × 10^5^ cells/well), stimulated with 10 ng/ml TNF-α, and concurrently treated with sSiglec-9 (0–10 ng/ml) for 12 h.

### Pretreatment with sialidase and CCR2 antagonist

To determine whether sialic acid and C-C chemokine receptor type 2 (CCR2) were required to induce the anti-inflammatory effects of sSiglec-9, we used sialidase [23] (Roche, Basel, Switzerland) to remove sialic acid and RS504393 (R&D Systems), a selective CCR2 antagonist, to inhibit the CCR2/CCL2 signaling pathway [24]. RAW264.7 cells were preincubated with 15 mU/300 μl sialidase for 1 h and 50 μM RS504393 for 20 minutes before stimulation.

### Real-time RT-PCR

Total RNA was extracted and subjected to real-time polymerase chain reaction (PCR) analysis to determine the messenger RNA (mRNA) expression of M1 macrophage markers (*TNF-α*, *IL-6*, and *iNOS*), M2 macrophage markers (*CD206*, *Arginase-1*, and *IL-10*), and *glyceraldehyde 3-phosphate dehydrogenase* (*GAPDH*) in RAW264.7 cells and pMACs. The mRNA expression of *matrix metalloproteinase* (*MMP*)*-1*, *MMP-3*, *MMP-13*, *IL-6*, and *GAPDH* was determined in FLS. The primer sequences (forward and reverse) used were as follows: *TNF-α*: 5′-TAGCCAGGAGGGAGAACAGA-3′ and 5′-TTTTCTGGAGGGAGATGTGG-3′; *IL-6* (mice): 5′-TTCCTTGGATTGGAGGTGAC-3′ and 5′-TGCCAGGAAAGGTTCTGAAG-3′; *iNOS*: 5′-TCCAGTTGCCTTCTTGGGAC-3′ and 5′-AATGGCGTGGAGCTGAGAGAT-3′; *CD206*: 5′-TCTCCCGGAACCGACTCTTC-3′ and 5′-AACTGGTCCCCTAGTGTACGA-3′; *Arginase-1*: 5′-GGTGGCAGAGGTCCAGAAGAA-3′ and 5′-GAGTGTTGATGTCAGTGTGAGCA-3′; *IL-10*: 5′-ATTTGAATTCCCTGGGTGAGAAG-3′ and 5′-CACAGGGGAGAAATCGATGACA-3′; *GAPDH *(mice): 5′- GGTCGGAGTCAACGGATTTG-3′ and 5′- ATGAGCCCCAGCCTTCTCCAT-3′; *MMP-1*: 5′-TTCCTTGGATTGGAGGTGAC-3′ and 5′-TGCCAGGAAAGGTTCTGAAG-3′; *MMP-3*: 5′-TTCCTTGGATTGGAGGTGAC-3′ and 5′-TGCCAGGAAAGGTTCTGAAG-3′; *MMP-13*: 5′-TTCCTTGGATTGGAGGTGAC-3′ and 5′-TGCCAGGAAAGGTTCTGAAG-3′; *IL-6* (human): 5′-TTCCTTGGATTGGAGGTGAC-3′ and 5′-TGCCAGGAAAGGTTCTGAAG-3′; *GAPDH* (human): 5′-TTCCTTGGATTGGAGGTGAC-3′ and 5′-TGCCAGGAAAGGTTCTGAAG-3′.

### ELISA and Western blot analysis

The effects of sSiglec-9 on the protein expression of M1 macrophage markers (TNF-α, IL-6, and iNOS) in RAW264.7 cells were evaluated by ELISA and Western blot analysis. RAW264.7 cells were treated with IFN-γ in the presence or absence of sSiglec-9 (0–20 ng/ml) for 24 h. Protein concentration was determined using the Bradford method (Bio-Rad Laboratories, Hercules, CA, USA). Extracted proteins were subjected to ELISA for TNF-α (BioLegend) and IL-6 (Takara Bio, Shiga, Japan) according to the manufacturer’s instructions. Since there were no commercially available ELISA kits for iNOS, we determined iNOS protein levels by Western blot analysis (40 μg/lane) using rabbit anti-iNOS and anti-β-actin antibodies (Cell Signaling Technology). We also examined phosphorylation levels of nuclear factor (NF)-kB and p38 by Western blot analysis using rabbit anti-phospho-NF-kB (p65), anti-total NF-kB (p65), anti-phospho-p38, and anti-total p38 antibodies as primary antibodies (Cell Signaling Technology). Cell lysates for the evaluation of NF-kB and p38 phosphorylation were obtained 20 minutes and 30 minutes after stimulation, respectively. The secondary antibody used for all Western blot analyses was an HRP-linked antirabbit IgG antibody (Cell Signaling Technology).

### Statistical analysis

Values are expressed as mean ± SEM. Statistical significance was analyzed using Student’s *t* test for two-group comparisons, analysis of variance (ANOVA) for multiple group comparisons, Pearson’s product-moment correlation coefficient (*R*) for relationships between pairs of parameters, and the χ^2^ test for comparisons of proportions. The significance of individual differences in the multiple group comparisons was evaluated using the Bonferroni post hoc test only if ANOVA indicated significance. *p* < 0.05 was considered significant. Statistical analyses were performed with IBM SPSS Statistics 20.0.0 for Windows software (IBM, Armonk, NY, USA).

## Results

### Evaluation of incidence and severity of arthritis

The results of in vivo imaging suggested that sSiglec-9 localized to inflamed tissue. The sites of accumulation of VT-labeled sSiglec-9 corresponded to inflamed extremities (Fig. 1a). The hind paws of control CIA mice (PBS-injected) showed severe swelling of the paws and all digits. The hind paws and digits of mice treated with low-dose (5 ng/g) sSiglec-9 showed mild swelling, whereas mice treated with high-dose (50 ng/g) sSiglec-9 showed no swelling (Fig. 1b).

Incidence rates and arthritis scores were evaluated from day 21 to day 42. Arthritis first developed on day 23 (i.e., 2 days after the second immunization). The cumulative incidence rate increased day by day and plateaued on day 38. Treatment of CIA mice with human sSiglec-9 reduced the cumulative incidence rate and severity (arthritis score) of arthritis in a dose-dependent manner (Fig. 1c and d). This beneficial effect was maximal in mice treated with 50 ng/g sSiglec-9. There was a significant difference between the control group and mice treated with 50 ng/g sSiglec-9 from day 37 to day 42 for incidence rate, and from day 36 to day 42 for the arthritis score.

### Serum concentration of TNF-α

The concentration of serum TNF-α significantly decreased in mice treated with sSiglec-9 compared with the control group in a dose-dependent manner (Fig. 1e). A strong positive correlation (*p* = 0.002, *R* = 0.837) was found between the arthritis score and serum concentration of TNF-α (Fig. 1f).

### Histological assessment of arthritis severity

**Fig. 2:**
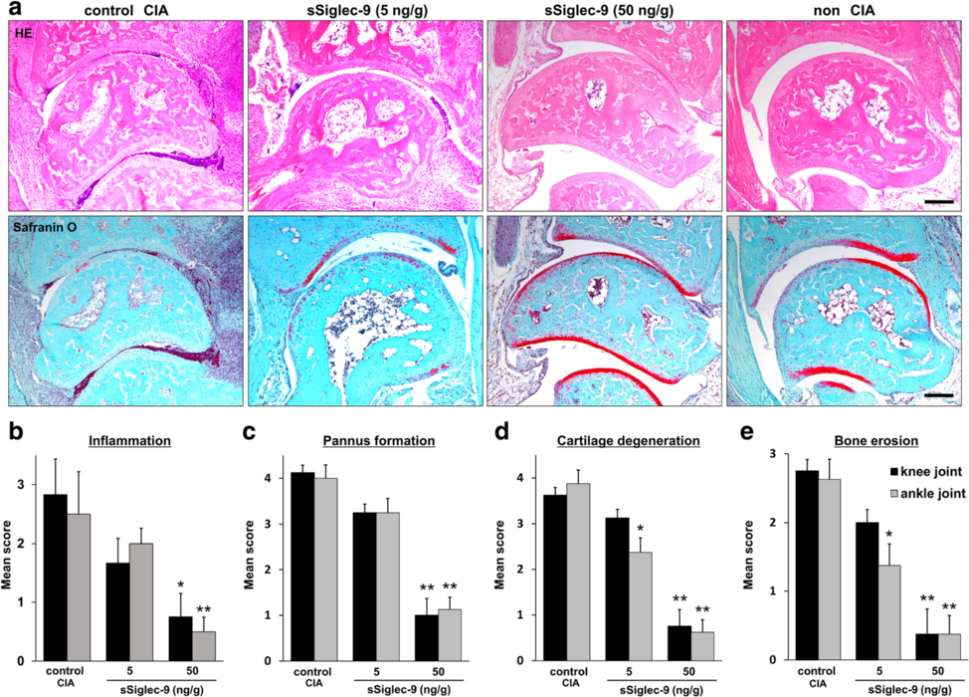
Histological analysis of inflamed joints in mice with collagen-induced arthritis (CIA). **a** The severity of arthritis was evaluated histologically in knee and ankle joint specimens obtained from control CIA mice (untreated) and mice treated with different dosages of soluble sialic acid-binding immunoglobulin-type lectin (sSiglec)-9. Each section was stained with hematoxylin and eosin (HE; *upper row*) or Safranin O (*lower row*). Serial sagittal sections (4.5 μm) were examined, and the maximally affected section was evaluated and scored. Typically, the midline of the medial condyle was examined. Scale bar = 100 μm. **b**–**e** Mean ± SEM histological arthritis scores (inflammation, pannus formation, cartilage degeneration, and bone erosion) in control CIA mice and mice treated with different dosages of sSiglec-9. *p < 0.05, ** p < 0.01 (Bonferroni post hoc test)

The ankle joints of control CIA mice exhibited histological changes, including synovial hyperplasia with a large number of infiltrating inflammatory cells, pannus formation, and severe cartilage and bone destruction. Although the ankle joints of mice treated with 5 ng/g sSiglec-9 showed mild synovitis and mild cartilage damage, those of mice treated with 50 ng/g sSiglec-9 showed no evident synovitis or obvious damage to cartilage or bone (Fig. 2a). sSiglec-9 significantly reduced the histological scores of all four parameters in both knee and ankle joints in a dose-dependent manner (Fig. 2b–e).

The effect of sSiglec-9 was remarkable in the scores of cartilage degeneration and bone erosion, and even the 5 ng/ml dosage of sSiglec-9 reduced the scores of these two parameters in the ankle joints.

### Immunohistochemical analysis of ankle specimens

**Fig. 3:**
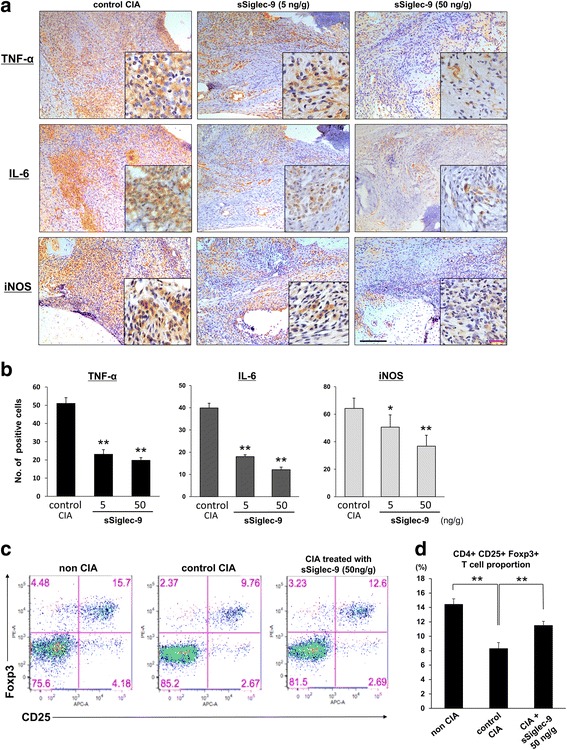
**a** Immunohistochemical staining of tumor necrosis factor (TNF)-α, interleukin (IL)-6, and inducible nitric oxide synthase (iNOS) in ankle joint specimens from control collagen-induced arthritis (CIA) mice and mice treated with different dosages of soluble sialic acid-binding immunoglobulin-type lectin (sSiglec)-9 at low (×200 original magnification) and high (×1000 original magnification; *insets*) magnifications. Scale bars: *black* = 100 μm; *red* = 20 μm. Strong positive staining was observed in control CIA mice, and weaker staining was observed in mice treated with sSiglec-9. **b** Immunopositive cells were counted individually in three different areas under a light microscope at × 400 original magnification. The number of positively stained cells was then averaged. The proportion of immunopositive cells was determined by dividing the number of positive cells by the total number of cells within the field. sSiglec-9 treatment significantly decreased the proportion of cells positive for TNF-α, IL-6, and iNOS. Values are presented as mean ± SEM. *p < 0.05, **p < 0.01 versus control CIA mice (Bonferroni post hoc test). **c** Regulatory T (Treg) cell expansion in isolated CD4^+^ splenocytes. Representative results of flow cytometric analysis of Treg cells are shown. CD4^+^CD25^+^Foxp3^+^ expansion in isolated splenocytes of normal mice, control CIA mice, and CIA mice treated with 50 ng/g sSiglec-9. **d** Isolated CD4^+^ T cells from splenocytes were subjected to intracellular staining of CD25 and forkhead box P3 (Foxp3) for flow cytometric analysis. The number of CD25^+^Foxp3^+^ T cells was significantly reduced in control CIA mice compared with normal mice. This reduction was significantly recovered in mice treated with sSiglec-9. **p < 0.01 (Bonferroni post hoc test)

Signal intensities for TNF-α, IL-6, and iNOS in synovial membranes decreased in groups treated with sSiglec-9 in a dose-dependent manner (Fig. 3a). The intensities of these proteins in control CIA mice were stronger than those in mice treated with sSiglec-9. The number of cells positive for TNF-α, IL-6, and iNOS was also significantly less in groups treated with sSiglec-9 than in control CIA mice (Fig. 3b).

### Normalization of Foxp3-positive regulatory T cells in mice treated with sSiglec-9

Flow cytometry revealed a reduced proportion of CD25^+^Foxp3^+^ regulatory T (Treg) cells in control CIA mice (8.3 ± 0.8 %) compared with the normal group (14.4 ± 0.8 %). CIA mice treated with sSiglec-9 (50 ng/g) showed a significant recovery of CD25^+^Foxp3^+^ Treg cells (11.5 ± 0.6 %) (Fig. 3c and d).

### sSiglec-9 reduces the levels of secreted M1 macrophage markers in RAW264.7 cells

**Fig. 4:**
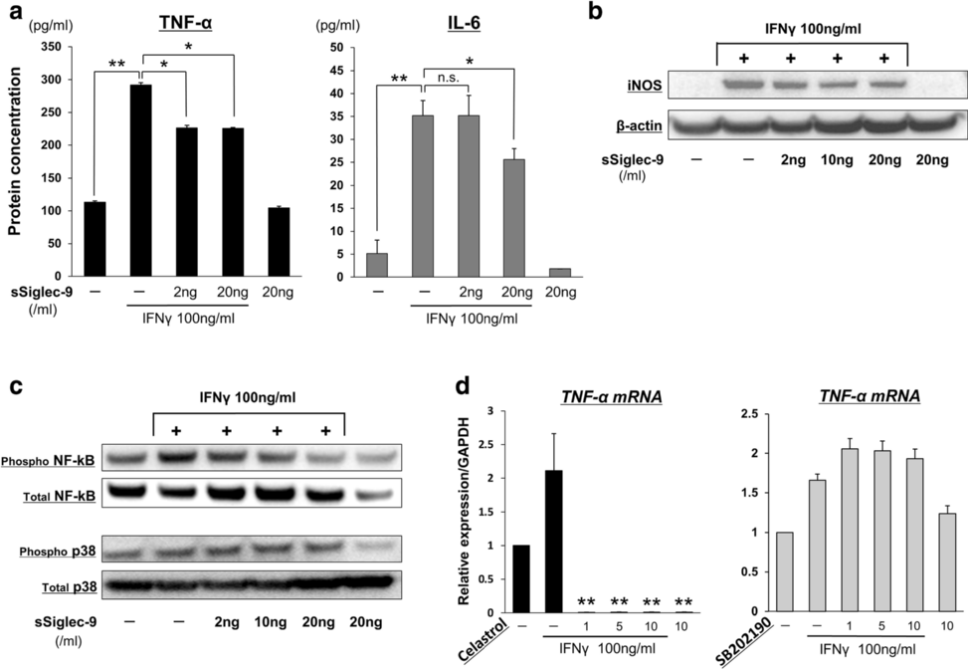
**a** and **b** Protein expression of tumor necrosis factor (TNF)-α and interleukin (IL)-6 was determined by enzyme-linked immunosorbent assay, and that of inducible nitric oxide synthase (iNOS) was determined by Western blot analysis following treatment with soluble sialic acid-binding immunoglobulin-type lectin (sSiglec)-9 (0–20 ng/ml) for 24 h. Expression of β-actin was used as a control. The expression of all M1 markers was significantly suppressed by sSiglec-9 treatment. *p < 0.05, **p < 0.01 (Bonferroni post hoc test). **c** Western blot analysis revealed that sSiglec-9 treatment dose-dependently decreased the interferon (IFN)-γ-stimulated phosphorylation of nuclear factor (NF)-kB p65, while the phosphorylation of p38 was not affected. **d** Celastrol, a specific inhibitor of NF-kB, significantly inhibited the IFN-γ-stimulated increase in TNF-α messenger RNA (mRNA) expression. However, SB202190, a specific inhibitor of p38/mitogen-activated protein kinase, did not affect TNF-α mRNA expression. **p < 0.01 versus samples stimulated with IFN-γ without sSiglec-9 treatment (Bonferroni post hoc test). *GAPDH* glyceraldehyde 3-phosphate dehydrogenase, *n.s.* not significant

The described experiments above clearly demonstrated the in vivo efficacy of sSiglec-9 in the CIA mice model. To determine whether these effects may be mediated by changes in secreted inflammatory cytokines, we assessed TNF-α, IL-6, and iNOS production in RAW264.7 cells stimulated with 10 ng/ml IFN-γ by ELISA and Western blot analysis. Treatment with sSiglec-9 significantly reduced the production of TNF-α, IL-6, and iNOS in a dose-dependent manner (Fig. 4a and b).

### Effect of sSiglec-9 on NF-kB and p38/MAPK signaling pathways

We next assessed the phosphorylation status of NF-kB (p65) and p38/mitogen-activated protein kinase (MAPK) to determine the signaling pathway inhibited by sSiglec-9. Treatment with sSiglec-9 significantly reduced the phosphorylation of NF-kB p65 in a dose-dependent manner, but it did not affect the phosphorylation of p38/MAPK (Fig. 4c). We also assessed the intracellular signaling pathways involved in the effects of sSiglec-9 and confirmed that phosphorylation of NF-kB p65 and p38/MAPK increased upon stimulation of RAW264.7 cells with IFN-γ (100 ng/ml).

Chemical inhibitors of NF-kB and p38/MAPK pathways were used to determine whether these pathways affected the IFN-γ-induced production of *TNF-α* mRNA. If NF-kB blockade does not reduce *TNF-α* production, there may be another pathway that sSiglec-9 inhibits in order to reduce *TNF-α* production. Pretreatment with celastrol, a specific NF-kB inhibitor, significantly suppressed *TNF-α* expression, even at the lowest dose tested (Fig. 4d). In contrast, pretreatment with SB202190, a specific p38 inhibitor, did not affect IFN-γ-induced *TNF-α* expression.

### Influence of sialidase and CCR2 antagonist on the effects of sSiglec-9

It was previously reported that sialic acid and CCR2 are required for sSiglec-9 to exert its anti-inflammatory effect [15]. Thus, we examined whether these two factors influenced the inhibitory effects of sSiglec-9 in IFN-γ-activated RAW264.7 macrophages. To this end, we added sialidase and the CCR2 inhibitor RS504393 to cells and examined *TNF-α*, *IL-6*, and *iNOS* mRNA levels as described above.

**Fig. 5:**
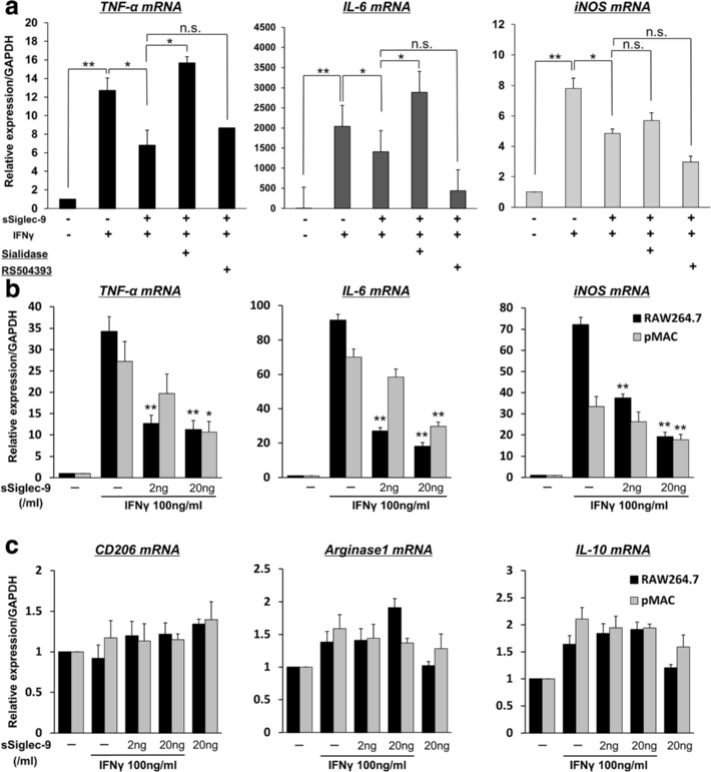
**a** Potential mechanism of action of soluble sialic acid-binding immunoglobulin-type lectin (sSiglec)-9 in murine macrophages. Sialidase was used to assess the effects of eliminating sialic acid from the cell surface on sSiglec-9 efficacy. sSiglec-9 treatment suppressed the messenger RNA (mRNA) expression of M1 markers. However, pretreatment with sialidase significantly blunted the suppressive effect of sSiglec-9, suggesting that sSiglec-9 required sialic acid to bind to its receptor. Pretreatment of cells with RS504393, a specific inhibitor for C-C chemokine receptor type 2 (CCR2), did not affect the suppressive effect of sSiglec-9, suggesting that CCR2 was not the primary receptor for sSiglec-9. *p < 0.05, **p < 0.01 (Bonferroni post hoc test). **b** Effects of sSiglec-9 on mRNA expression of M1 macrophage markers in RAW264.7 cells and peritoneal macrophages (pMACs). mRNA expression of M1 macrophage markers (*tumor necrosis factor *[*TNF*]-*α*, *interleukin* [*IL*]-*6*, and *inducible nitric oxide synthase* [*iNOS*]) was determined by real-time polymerase chain reaction (RT-PCR). Cells were stimulated with interferon (IFN)-γ in the presence or absence of sSiglec-9 (0–20 ng/ml) for 12 h. Expression of *glyceraldehyde 3-phosphate dehydrogenase* (*GAPDH*) mRNA was used as a control. mRNA expression of all M1 markers was significantly suppressed by sSiglec-9 treatment in both cell types. *p < 0.05, **p < 0.01 versus samples stimulated with IFN-γ without sSiglec-9 treatment (Bonferroni post hoc test). **c** Effects of sSiglec-9 on mRNA expression of M2 macrophage markers in RAW264.7 cells and pMACs. mRNA expression of M2 macrophage markers (*CD206*, *Arginase-1*, and *IL-10*) was determined by RT-PCR under the same conditions as described above for M1 markers. mRNA expression of all M2 markers did not significantly change with sSiglec-9 treatment in both cell types (Bonferroni post hoc test). *n.s.* not significant

Pretreatment with sialidase significantly blunted the inhibitory effect of sSiglec-9 on IFN-γ-stimulated mRNA expression of *TNF-α* and *IL-6*. This suggested that sSiglec-9 exerted its anti-inflammatory effect by binding to sialic acid (Fig. 5a). Pretreatment with RS504393 did not affect the inhibitory effect of sSiglec-9 on IFN-γ-stimulated mRNA expression of M1 markers (Fig. 5a). However, CCR2 blockade reduced *IL-6* and *iNOS* mRNA expression relative to that in the sSiglec-9-treated groups. These findings suggest that CCR2 is unlikely to be involved in the mechanism of action of sSiglec-9. This finding differs from that of a previous study in a rat SCI model [15]. These results suggested that concomitant CCR2/CCL2 blockade may augment the anti-inflammatory effects of sSiglec-9 in the CIA mouse model.

Taken together, our results suggest that sSiglec-9 required sialic acid on the cell surface to exert its anti-inflammatory effect. In contrast to the rat SCI model, CCR2 was not a receptor for sSiglec-9 in the mouse CIA model.

### sSiglec-9 reduces the expression of M1 markers, but not M2 markers, in RAW264.7 cells and pMACs

Considering potential differences between macrophage cell lines and primary macrophages, the macrophage cell line RAW264.7 and pMACs were treated with IFN-γ in the absence or presence of sSiglec-9 in vitro. Moreover, to determine whether sSiglec-9 exerts an inhibitory effect on inflammatory macrophage function, RT-PCR was performed to assess mRNA expression of M1 markers (*TNF-α*, *IL-6*, and *iNOS*) and M2 markers (*CD206*, *Arginase-1*, and *IL-10*).

The expression of M1 markers (*TNF-α*, *IL-6*, and *iNOS*) in RAW264.7 cells and pMACs was significantly suppressed by coculture with sSiglec-9 (Fig. 5b). The inhibitory effect of sSiglec-9 was substantial in RAW264.7 cells, especially with respect to *IL-6* expression. sSiglec-9 did not significantly affect M2 marker expression in both cell types, although only *Arginase-1* and *IL-10 *expression in pMACs was increased with IFN-γ stimuli (Fig. 5c).

### Effect of sSiglec-9 is not observed in human FLS derived from patients with RA

Synovial tissues in patients with RA consist of synovial macrophages and FLS. MMPs and IL-6 are produced by FLS and play a major role in the destruction of cartilage in RA joints [25, 26]. Therefore, we also examined the inhibitory effect of sSiglec-9 on FLS stimulated with TNF-α and evaluated the expression of *MMP-1*, *MMP-3*, *MMP-13*, and *IL-6* using RT-PCR.

**Fig. 6:**
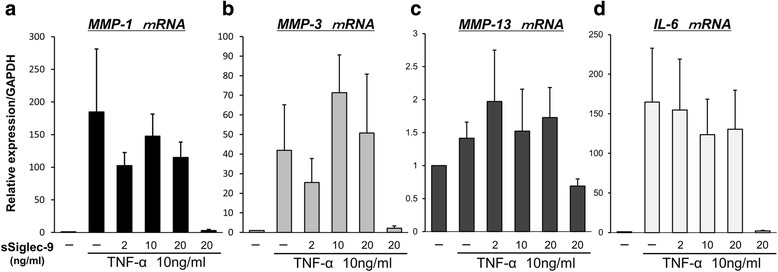
Effect of soluble sialic acid-binding immunoglobulin-type lectin (sSiglec)-9 on the messenger RNA (mRNA) expression of *matrix metalloproteinase *(*MMP*)*-1*, *MMP-3*, *MMP-13*, and *interleukin* (*IL*)*-6* in fibroblast-like synoviocytes (FLS) derived from synovial tissue of patients with rheumatoid arthritis. FLS were stimulated with tumor necrosis factor (TNF)-α in the presence or absence of sSiglec-9 (0–20 ng/ml) for 12 h. Relative mRNA expression of *MMP-1*, *MMP-3*, *MMP-13*, and *IL-6* standardized using *glyceraldehyde 3-phosphate dehydrogenase *(*GAPDH*) was determined by real-time polymerase chain reaction. **a, b, c, d** sSiglec-9 treatment had no effect on the TNF-α-stimulated induction of *MMP-1*, *MMP-3*, *MMP-13*, and *IL-6* mRNA expression

Contrary to the macrophage data described above, TNF-α-stimulated mRNA expression levels of *MMP-1*, *MMP-3*, and *MMP-13*, as well as of *IL-6*, were not affected by sSiglec-9 treatment in human FLS (Fig. 6a-d ). These data suggest that synovial cells are unlikely to be involved in the effects of sSiglec-9 and that the beneficial effects of sSiglec-9 can be attributed to its effects on macrophages.

## Discussion

Treatment strategies for RA have improved dramatically over the past decade. TNF inhibitors comprise an important aspect of current RA treatment, and their therapeutic effects were first confirmed in the CIA model, an established animal model of RA [27]. In this study, we found that treatment of CIA mice with sSiglec-9 suppressed arthritis symptoms in vivo and had a suppressive effect on M1 macrophage activation in vitro. New antirheumatic agents with novel mechanisms of action are likely to arise, given that patients with RA can become resistant to multiple preexisting drugs. In this context, sSiglec-9 could be a new option for such patients.

Treatment with sSiglec-9 effectively suppressed principal indices of arthritis in the CIA model. Specifically, the incidence and severity of CIA were reduced, as was the serum concentration of TNF-α. Importantly, treatment with sSiglec-9 significantly recovered the reduced proportion of Foxp3^+^ Treg cells in CIA mice. Foxp3^+^ Treg cells are mediators of autoimmune reaction inhibition, and a reciprocal relationship between the differentiation of Th17 and Treg cells has been reported [28]. This suggests that sSiglec-9 may be an effective treatment option for RA by regulating the balance of Treg and Th17 cell differentiation.

Exposure to a drug at the site of arthritis is an important consideration for the effective treatment of RA. In this study, we found that intravenously injected sSiglec-9 localized mainly to inflamed joints. In a previous study, the distribution of TNF-α inhibitors (certolizumab pegol, adalimumab, and infliximab) in CIA mice was evaluated using a biofluorescence method similar to ours. In that study, all TNF-α inhibitors localized to inflamed tissue, mainly within the limbs [29]. We observed enhanced penetration of sSiglec-9 into inflamed tissue, as with the TNF-α inhibitors, and demonstrated the relevance of using sSiglec-9 as a therapeutic agent to treat RA.

In the SCI model [15], sSiglec-9 alters macrophage polarization from M1- to M2-dominant by modifying the intracellular CCR2 signaling pathway. The M2-dominant polarization of macrophages reduces tissue damage, preserves descending 5-hydroxytryptamine-positive axons, and promotes functional recovery after SCI. M1 macrophages are involved in acute inflammation, and their classical activation pathway is activated mainly by IFN-γ and LPS. M1 macrophages play many important roles in the early phase of innate immunity and function as effector cells in Th1 cellular immune responses, producing large amounts of TNF-α, IL-12, IL-23, and iNOS [30]. In the experiments using RAW264.7 cells and pMACs, sSiglec-9 effectively suppressed the expression of M1 markers. There was no apparent difference between cell lines and primary macrophages in response to sSiglec-9 treatment. However, unlike in the SCI model, M2 macrophage markers did not increase with sSiglec-9 treatment. Thus, the effects of sSiglec-9 differ across diseases and animal models, and sSiglec-9 suppresses the activation of M1 macrophages in the CIA mice model.

The mechanism of action of sSiglec-9 and its receptor are unknown. In a study by Matsubara et al. [15], the depletion of CCR2, treatment with a selective CCR2 inhibitor, and the genetic knockout of CCR2 abolished the effects of sSiglec-9 on bone marrow-derived macrophages. In our in vitro model, however, CCR2 blockade did not abolish the effects of sSiglec-9. Thus, the primary receptor for sSiglec-9 is unlikely to be CCL2 in macrophages. Instead, CCR2 blockage appears to augment the suppression of IFN-γ-induced M1 marker expression. CCL2, a ligand for CCR2, is an inflammatory chemokine that is highly present in the serum of patients with RA relative to the serum of those with osteoarthritis [31], and a positive correlation has been reported between serum CCR2 concentration and RA disease activity [32]. Thus, inhibition of the CCR2/CCL2 pathway may more effectively suppress M1 macrophage activity and disease activity in CIA. Further studies are needed to identify the primary receptor for sSiglec-9 and the contribution of the CCR2/CCL2 pathway in the pathogenesis of CIA.

Matsubara et al. [15]’s work also suggests that sSiglec-9 binds sialylated carbohydrates on CCR2, inducing an M2 shift, which can be prevented by removing sialic acid with sialidase. Consistent with this, sialidase abolished the ability of sSiglec-9 to inhibit stimulated macrophages. These results underscore the importance of the binding of sSiglec-9 to its receptor via sialic acid, although this receptor has yet to be identified in the CIA model.

In the present study, IFN-γ was used to stimulate RAW264.7 cells and pMACs. This cytokine has a strong inflammatory effect and elicits an immune response in mammals. It has a wide range of activities in distinct cell populations, including both immune and nonimmune cells. For instance, IFN-γ-primed macrophages exhibit increased migration mediated by the CCR2/CCL2 pathway. IFN-γ-induced, signal transducer and activator of transcription 1 (STAT1)-independent pathways result in the activation of phosphoinositide 3-kinase, Akt, p38, protein kinase C, inhibitor of kB kinases, and STAT3, and they have important functions both in vitro and in vivo, including the promotion of inflammation and cell proliferation [33]. In the present study, sSiglec-9 did not suppress p38 phosphorylation, but it significantly suppressed NF-kB phosphorylation. Additionally, pretreatment with celastrol, a selective NF-kB inhibitor, almost completely suppressed the IFN-γ-mediated induction of TNF-α mRNA expression. The NF-kB signaling pathway is involved in the pathogenesis of inflammatory arthritis, including RA [22]. Collectively, these findings suggest that sSiglec-9 effectively suppresses M1 macrophage activation by downregulating the NF-kB pathway.

Murine Siglec-E, which corresponds to human Siglec-9, inhibits Toll-like receptor (TLR)-induced NF-kB, and negatively regulates TLR responses, including the downstream production of TNF-α and IL-6. The expression of Siglec-E can be induced by LPS or IFN-β (but not IFN-γ) [34]. In one report, Siglec-H (also murine CD33-related Siglec) was found to be upregulated by IFN-γ in microglia, with polarization shifting to M1 [35]. These data imply that sSiglec-9 may bind to a TLR-related receptor induced by IFN-γ and downregulate NF-kB activity.

Synovial tissues in patients with RA consist of synovial macrophages and FLS. FLS are also considered a key cell type that mediates the destruction of cartilage and bone in affected joints [36]. Activation of local FLS is driven by both proinflammatory cytokines and cytokine-independent pathways, including endogenous retroviral elements and TLRs [37]. We examined the expression of MMP-1, MMP-3, MMP-13 and IL-6, since FLS are the primary source of MMP expression in RA. MMPs are structurally and functionally related enzymes and are subdivided into five groups depending on their matrix protein substrates. Collagenases (MMP-1 and MMP-13) and stromelysin (MMP-3) are important for the degradation of cartilage in RA. Their synthesis and activation are induced by various factors, including proinflammatory cytokines and TLR ligands [38, 39].

sSiglec-9 did not exert its beneficial effects on FLS stimulated with TNF-α. Thus, the inhibitory effect of sSiglec-9 likely occurs via the suppression of M1 macrophages, but not local FLS activation, implying that sSiglec-9 may regulate systemic immune responses in inflammatory diseases.

There are some limitations of this study. First, sSiglec-9 had an anti-inflammatory effect in macrophages, but not in FLS, in vitro. It is possible that other immune cells, such as neutrophils or T cells other than macrophages, are targets of sSiglec-9. This possibility, as well as the receptor for sSiglec-9 and downstream signaling pathways, needs to be assessed in future studies. Second, we did not assess the safety profile of sSiglec-9 in this study. sSiglec-9 may have an immunosuppressive effect, given its ability to suppress macrophage activity. Therefore, infectious diseases or neoplasms are a possibility with sSiglec-9 therapy. However, none of the animals used in this study had an infectious disease or neoplasm after treatment with sSiglec-9, although the treatments were short-term. Further studies are needed to assess the long-term safety profile of sSiglec-9 before its use in humans.

## Conclusions

sSiglec-9 significantly reduced the incidence and severity of arthritis in a CIA mouse model. It also reduced M1 macrophage activation via NF-kB suppression in vitro. Given that many patients do not adequately respond to currently available biologics, this new agent could contribute to RA treatment strategies via a new mechanism of action.

## Abbreviations

ANOVA, analysis of variance; CCL2, chemokine (C-C motif) ligand 2; CCR2, C-C chemokine receptor type 2; CFA, complete Freund’s adjuvant; CIA, collagen-induced arthritis; DMARD, disease-modifying antirheumatic drug; ELISA, enzyme-linked immunosorbent assay; FLS, fibroblast-like synoviocyte(s); Foxp3, forkhead box P3; GAPDH, glyceraldehyde 3-phosphate dehydrogenase; HE, hematoxylin and eosin; IFN-γ, interferon-γ; Ig, immunoglobulin; IL, interleukin; iNOS, inducible nitric oxide synthase; LPS, lipopolysaccharide; MAPK, mitogen-activated protein kinase; MCP-1, monocyte chemoattractant protein 1; MMP, matrix metalloproteinase; mRNA, messenger RNA; NF-kB, nuclear factor-kB; pMAC, peritoneal macrophage; RA, rheumatoid arthritis; RT-PCR, real-time polymerase chain reaction; SCI, spinal cord injury; Siglec-9, sialic acid-binding immunoglobulin-type lectin 9; sSiglec-9, soluble sialic acid-binding immunoglobulin-type lectin 9; STAT, signal transducer and activator of transcription; TLR, Toll-like receptor; TNF-α, tumor necrosis factor-α; Treg, regulatory T (cell); VT, VivoTag
